# Foveolar gastric metaplasia presenting as a duodenal tumor with an atypical appearance: a case report

**DOI:** 10.1186/s13256-016-1163-5

**Published:** 2016-12-20

**Authors:** Takahiro Abe, Masayuki Kato, Shuzo Kohno, Sigeharu Hamatani, Yosuke Kawahara, Kimio Isshi, Koji Matsuda, Kazuki Sumiyama

**Affiliations:** 1 Department of Endoscopy, The Jikei University Katsushika Medical Center, 6-41-2 Aoto Katsushika-ku, Tokyo, 125-8506 Japan; 2 Department of Surgery, The Jikei University Katsushika Medical Center, 6-41-2 Aoto Katsushika-ku, Tokyo, 125-8506 Japan; 3 Department of Pathology, Clinical Service, The Jikei University Katsushika Medical Center, 6-41-2 Aoto Katsushika-ku, Tokyo, 125-8506 Japan; 4 Department of Endoscopy, The Jikei University School of Medicine, 3-25-8 Nishishinbashi Minato-ku, Tokyo, 105-8461 Japan

**Keywords:** Foveolar gastric metaplasia (FGM), Ectopic gastric mucosa, Narrow band imaging (NBI), Endoscopic ultrasonography (EUS)

## Abstract

**Background:**

Foveolar gastric metaplasia of the duodenum is a frequent but not as yet considered correlate of endoscopically detected duodenal polyps. The majority of foveolar gastric metaplasias associated with polyps presented a typical benign endoscopic appearance and they were diagnosed by biopsy. Here we report a case of a surgical-resected foveolar gastric metaplasia manifesting as a duodenal tumor with an atypical appearance.

**Case presentation:**

An asymptomatic 56-year-old Asian man who presented with a foveolar gastric metaplasia of atypical appearance and had previously undergone esophagogastroduodenoscopy was referred to our hospital. A biopsy revealed a normal duodenum with an inflamed mucosa. Narrow band imaging with magnifying endoscopy revealed normal microvessels with normal micromucosa, which indicated non-neoplasia. Endoscopic ultrasonography using a miniature probe system (20 MHz) revealed a hypoechoic mass with multiple anechoic lesions (16-mm diameter) located in the mucosal layer. The lesion was excised via laparotomy assisted by endoscopic techniques similar to endoscopic submucosal dissection. The pathology indicated foveolar gastric metaplasia.

**Conclusions:**

Foveolar gastric metaplasia can present as a duodenal tumor. We identified two important clinical issues. First, foveolar gastric metaplasia can present as a duodenal tumor with an atypical benign appearance. Second, both endoscopic ultrasonography and narrow band imaging are useful techniques to increase the diagnostic rate of this condition.

## Background

Foveolar gastric metaplasia (FGM) of the duodenum is a frequent but not as yet considered correlate of endoscopically detected duodenal polyps. According to a comparison of findings from two institutes with upwards of 100,000 gastroenterological cases per year, the association of FGM with typical polyp morphology was shown to be at higher rates than previously identified [1]. Results from this comparison showed a 41% association in one institute and 32% in the other institute. The majority of cases of FGM associated with polyps presented a typical benign endoscopic appearance and they were diagnosed by biopsy. Here we report a case of a surgical-resected FGM manifesting as a duodenal tumor with an atypical appearance.

## Case presentation

**Fig. 1:**
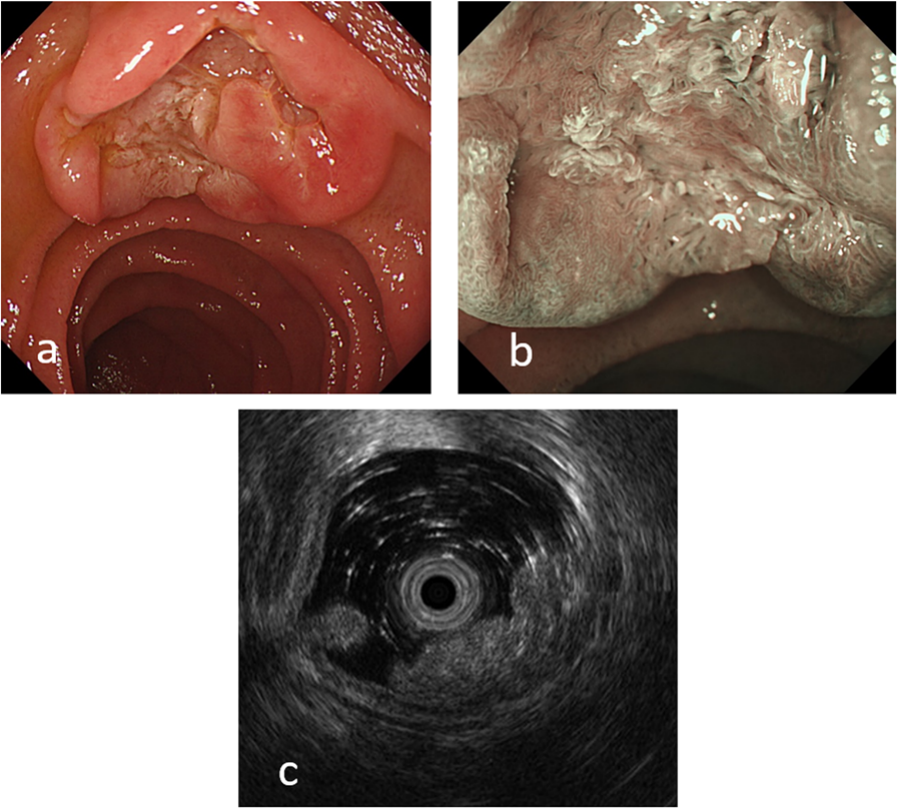
**a** An endoscopic picture showing a wide-based sessile submucosal tumor-like mass with depression at the top of the lesion. **b** Narrow banding image with magnification showing non-irregular microvessel or mucosal structure. **c** Endoscopic ultrasound images showing a heterogeneously hyperechoic mass with several anechoic lesions located predominantly in the mucosa

**Fig. 2:**
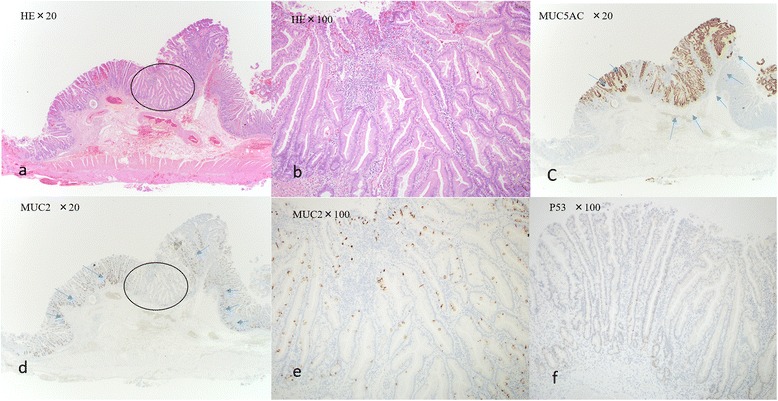
**a** Photomicrograph of the resected duodenal specimen shows irregular sequence of the tubular epithelium; hematoxylin and eosin (H&E), original magnification, ×20. **b** Hematoxylin and eosin (original magnification, ×100). **c** Immunohistological staining of foveolar epithelium for mucin 5AC (MUC5AC). No staining of the original duodenal epithelium. Brown cytoplasmic staining of the foveolar gastric metaplasia (*blue arrows*; magnification, ×20). **d** Immunohistological staining of goblet cell in duodenum mucin 2 (MUC2). No staining of the lesion. Brown staining of the original duodenal epithelium (magnification, ×20). **e** Mucin 2 (magnification, ×100). **f** Immunohistological staining of a malignant potential for p53. No staining of p53 (magnification, ×100). The circled within the panel denotes high power of H & E stained slide (a→b) and MUC2 (d→e)

An asymptomatic 56-year-old Asian man who presented with an FGM of atypical appearance and had previously undergone esophagogastroduodenoscopy was referred to our hospital. He had taken antihypertensive medicine for hypertension. He denied alcohol intake and did not smoke tobacco. An abdominal observation revealed that his abdomen was soft and flat and without pain. His neurological findings were normal. The results of laboratory findings were normal (Table 1). A biopsy revealed a normal duodenum with an inflamed mucosa. The tissue proximal to the esophagogastroduodenoscopy showed a wide-based sessile submucosal tumor-like mass. A 2-cm depression on top of the lesion was observed in the anterior wall of the second part (descending) of his duodenum. A biopsy was performed and repeated, with both results showing no malignancy. Narrow band imaging (NBI) with magnifying endoscopy revealed normal microvessels with normal micromucosa, which indicated non-neoplasia. Endoscopic ultrasonography (EUS) using a miniature probe system (20 MHz) revealed a hypoechoic mass with multiple anechoic lesions (16-mm diameter) located in the mucosal layer (Fig. 1). Although the evidence suggested that the lesion was benign, other causes including lymphoma, ectopic pancreatic tissue, carcinoid, or leiomyosarcoma of the duodenum were considered. Therefore, the lesion was excised via laparotomy assisted by endoscopic techniques, similar to endoscopic submucosal dissection (ESD). The pathology indicated FGM without gastric glands (Fig. 2). He was discharged from our hospital 13 days after surgery without postoperative complications.

**Table 1:** The result of laboratory findings

White blood cell count (μl)	8300
Red blood cell count (10^3^/μl)	506
Hemoglobin (g/dl)	15.7
Aspartate transaminase (U/L)	20
Alanine transaminase (U/L)	29
Alkaline phosphatase (U/L)	157
Total protein (g/dL)	7.2
Creatinine (mg/dl)	0.66
Sodium (Na; mmol/L)	138
Potassium (K; mmol/L)	4.6
C-reactive protein (mg/dl)	0.1
Carcinoembryonic antigen (CEA; ng/ml)	4.3
Cancer antigen (CA19-9; U/ml)	7

The esophagogastroduodenoscopy showed no recurrence of the lesion after 10 months.

## Discussion

We identified two important clinical issues: FGM can present as a duodenal tumor with an atypical benign appearance, and both EUS and NBI are useful techniques to increase the diagnostic rate of this condition.

First, FGM can present as a duodenal neoplasia with an atypical benign appearance. FGM is common in the duodenum, with localized ectopic gastric mucosa [2]. A search of the PubMed and Medline databases, at the Jikei University Library of Medicine, using the keyword “ectopic (heterotopic) gastric mucosa duodenum” or “foveolar gastric metaplasia duodenum” identified two reports that indicated that resections were performed to rule out malignancies in ectopic gastric mucosa with an abnormal appearance [3, 4]. With particular regard to FGM, the present case study is the first to differentiate between malignant and benign cases. For this case, a decision was made to resect the lesion via endoscopy-assisted laparotomy. After making circumferential endoscopic incisions, similar to a technique used in ESD, the lesion was surgically excised via laparotomy.

Second, both EUS and NBI were useful to increase the diagnostic rate of FGM. According to the validity report for EUS for ectopic gastric mucosa lesions, endoscopic resection may be used in cases of duodenal ectopic gastric mucosa when EUS can verify that the tumor is localized in the third layer, which corresponds to the submucosa of the duodenal wall [3]. In this case, EUS results verified that the lesion was located in the mucosal layer and was demonstrated to be a heterogeneously hypoechoic mass with small anechoic areas (Fig. 1). A few reports have described the diagnostic value of NBI in duodenal endoscopy [5]. In the present case, using NBI, we ruled out duodenal tumor because of the absence of irregular microvessel and micromucosal patterns. Multiple biopsy results supported the NBI findings that the lesion was not malignant.

In our case, although the lesion was diagnosed as benign, surgical resection was scheduled because of the relatively large size and unusual morphology of the lesion. We must concede that because the lesion was identified as benign, an observation period could have been an acceptable alternative. However, multiple factors led us to proceed with our chosen course of action, and valuable information was obtained.

## Conclusions

FGM can present as a duodenal tumor for which both EUS and NBI are valuable diagnostic tools. FGM can present as a duodenal tumor with an atypical benign appearance, and this should be considered while treating patients in future. In fact, many occurrences may remain unrecognized and undetected. EUS and NBI are highly recommended to verify and diagnose FGM before resection is considered. Further reporting would be useful to support the evidence that the incidence of FGM may be higher than previously thought, resulting in undiagnosed and untreated cases.

## Abbreviations

ESD: Endoscopic submucosal dissection
EUS: Endoscopic ultrasonography
FGM: Foveolar gastric metaplasia
NBI: Narrow band imaging
